# Heparan sulphate inhibition of cell proliferation induced by TGFβ and PDGF

**DOI:** 10.1155/S0962935193000419

**Published:** 1993

**Authors:** lan E. Silber, Jeanine M. Walenga, Jawed Fareed, Elizabeth J. Kovacs

**Affiliations:** 1 Molecular Biology Program Neurobiology and Anatomy Loyola University Maywood IL 60153 USA; 2 Dept of Thoracic and Cardiovascular Surgery Neurobiology and Anatomy Loyola University Maywood IL 60153 USA; 3 Dept of Pharmacology Neurobiology and Anatomy Loyola University Maywood IL 60153 USA; 4 Dept of Cell Biology Neurobiology and Anatomy Loyola University Maywood IL 60153 USA

## Abstract

The effect of glycosaminoglycans (GAGs) on the proliferation of smooth muscle cells (SMC) and fibroblasts was assessed by culturing cells with or without GAGs. Porcine heparan sulphate (HS) inhibited proliferation in a dose dependent manner. At 167 μg/ml of HS this reached 88% and 72% inhibition of SMC and fibroblast growth, respectively. Pig and beef mucosal heparins also blocked proliferation, but to a lesser extent. In contrast, beef lung heparin, chondroitin sulphate, and dermatan sulphate failed to block growth factor induced proliferation. Continuous presence of HS was not required, suggesting that the inhibitory effects resulted from a direct effect on the cell rather than an interaction of the GAG with growth factors. The mechanism by which GAGs inhibit proliferation will be addressed in future studies.


Mediators of Inflammation 2, 299–302 (1993)

### Heparan sulphate inhibition of cell proliferation induced by TGFβ and PDGF

Ian E. Silber,[1] Jeanine M. Walenga,[2,3] Jawed Fareed,[3] and Elizabeth J. Kovacs[1,4,CA]

[1]Molecular Biology Program, [2] Dept of Thoracic and Cardiovascular Surgery, [3] Dept of Pharmacology and [4] Dept of Cell Biology, Neurobiology and Anatomy, Loyola University, Maywood, IL 60153, USA

[CA] Corresponding Author

**THE effect of glycosaminoglycans (GAGs) on the proliferation of smooth muscle cells (SMC) and fibroblasts was assessed by culturing cells with or without GAGs. Porcine heparan sulphate (HS) inhibited proliferation in a dose dependent manner. At 167 μg/ml of HS this reached 88% and 72% inhibition of SMC and fibroblast growth, respectively. Pig and beef mucosal heparins also blocked proliferation, but to a lesser extent. In contrast, beef lung heparin, chondroitin sulphate, and dermatan sulphate failed to block growth factor induced proliferation. Continuous presence of HS was not required, suggesting that the inhibitory effects resulted from a direct effect on the cell rather than an interaction of the GAG with growth factors. The mechanism by which GAGs inhibit proliferation will be addressed in future studies.**

**Key words:** Fibroblast, Glycosaminoglycans, Growth factor, Proliferation, Smooth muscle cell

### Introduction

Heparin has been reported to inhibit the growth of cells *in vitro*.[1–5] However, due to its anticoagulant properties its value as a therapeutic agent in preventing the aberrant cell proliferation, which is the hallmark of a variety of diseases such as atherosclerosis and pulmonary fibrosis, is severely glycosaminoglycans limited. This limitation would not apply for (GAGs) such as heparan sulphate (HS) which are not very strong anticoagulants.[6]

Glycosaminoglycans are linear anionic polyelectrolytes composed of alternating glucosamine and uronic acid sugars.[7] HS, for example, contains uronic acids that are mostly glucuronic with small amounts of iduronic present, the glucosamine units are largely N-acetylated with a small number being N-sulphated. In heparin, the uronic acids differ from those found in HS, as there is more iduronic acid than glucuronic, also the glucosamine pattern is reversed such that the extent of N-sulphation is largely increased over the N-acetylation.[6]

To determine whether GAGs other than heparin can prevent cell proliferation, a series of GAGs including HS, dermatan sulphate, and chondroitin sulphate, were examined and tested for their ability to inhibit the replication of mesenchymal cells triggered by growth factors. Heparin was used for comparative purposes. The studies reveal that a porcine HS with low anticoagulant activity had a significant inhibitory effect on growth factor induced proliferation of Nor-10 smooth muscle cells (SMC), NIH 3T3 fibroblasts, and U-2 human osteosarcoma cells. The continuous presence of HS was not necessary to prevent the growth factor induced proliferation of cells. This suggested that the GAG was acting directly on the target cell, rather than merely binding to, and removing, growth factors in the media.

### Materials and Methods

*Reagents:* GAGs used in these studies were obtained from the following sources: porcine mucosal heparin (PMH) from Institut Choay (Paris, France); bovine mucosal heparin (BMH), porcine pancreatic heparan sulphate (HS), and bovine mucosal dermatan sulphate (DS) from Opocrin S.p.A. (Modena, Italy); bovine lung heparin (BLH) from the Upjohn Company (Kalamazoo, MI); and bovine trachea chondroitin sulphate C (CS) from The Sigma Company (St Louis, MO). Human platelet derived growth factor (PDGF) and porcine transforming growth factor-β (TGFβ) were obtained from R and D Systems (Minneapolis, MN). All tissue culture reagents were obtained from GIBCO Laboratories (Grand Island, NY).

*Cell culture and proliferation assay:* Proliferation assays were performed with a murine smooth muscle cell line (Nor-10), murine NIH 3T3 fibroblasts and a human osteosarcoma cell line (U-2). Nor-10 and U-2 cells were obtained from ATCC (Rockville, MD); NIH 3T3 cells were the kind gift of Dr Howard Young (NCI, Frederick, MD). Cells were cultured in Dulbecco's Modified Eagle's Medium (DME) with 5% foetal bovine serum (FBS), penicillin (100 units/ml), streptomycin (100 μg/ml)

© 1993 Rapid Communications of Oxford Ltd and glutamine (2 mM) in an atmosphere of 5% $CO_2$ in air at 37°C.

The cell proliferation assay used in these studies is a simple, reproducible, non-radioactive assay modified as follows from Kamijo *et al.*[8] Cells were plated in 96-well plates at a density of 4200 cells per well in DME supplemented with 1% foetal bovine serum (FBS). After 18 h, GAGs were added in triplicate in medium containing 10% FBS or purified growth factors (PDGF at 5 ng/ml or TGFβ at 15 ng/ml). Medium containing 10% FBS, without GAGs, and medium without FBS served as positive and negative controls, respectively. After 72 h cells were stained with crystal violet, solubilized with SDS, and the optical density (O.D.) was measured with a Dynatech (Chantilly, VA) ELISA plate reader. The maximal O.D. for cells grown in the presence of medium containing 10% FBS ranged from 0.3 to 1.5. The O.D. for freshly plated cells was comparable to that of cells cultured with growth inhibitory GAGs, ranging from 0.02 to 0.07. Percent inhibition was calculated as 1 − (O.D. lysate of cells treated with growth factors and GAGs/O.D. lysate of cells treated with growth factors alone) × 100. All experiments were repeated a minimum of three times. The increase in O.D. values is not due to an increase in cell size but in actual cell number as counted by a haemacytometer in response to various concentrations of FBS.

To determine whether the continuous presence of the GAG was necessary for inhibition of cell proliferation, cells were cultured for 18 h with GAGs, after which the medium was replaced with medium containing 10% FBS, 15 ng/ml TGFβ or 5 ng/ml PDGF. Cells were incubated for an additional 48 h, after which time they were stained and O.D. determined.

### Results

*Evaluation of the effect of GAGs on SMC proliferation:* A subset of GAGs was tested for their effects on SMC proliferation (Table 1). Of the GAGs tested, HS caused the greatest inhibition of growth followed by PMH and BMH. At 167 μg/ml, HS inhibited 88% of the growth factor activity in 10% FBS, whereas PMH and BMH inhibited growth by 72% and 41%, respectively. In contrast, BLH, CS, and DS failed to diminish the growth factor induced proliferation of SMC (data not shown). HS exhibited dose dependent inhibition of SMC growth (Fig. 1). Half maximal inhibition of proliferation was noted at 35–40 μg/ml. Since HS and PMH caused the greatest amount of growth inhibition, subsequent studies focused on these GAGs. HS consistently has a greater inhibitory effect on SMC proliferation than PMH even at levels as low as 20 μg/ml. The effects of GAGs on

**Table 1.** Effect of GAGs on the proliferation of Nor-10 SMC, NIH 3T3 fibroblasts and U2 osteosarcoma cells

| Treatment[b] | Cell number[a] | |
|---|---|---|
| | O.D. ± S.D. | Inhibition |
| **Nor-10 cells** | | |
| No GAG | 0.70 ± 0.04 | – |
| HS | 0.09 ± 0.02 | 88% |
| PMH | 0.20 ± 0.01 | 72% |
| BMH | 0.42 ± 0.03 | 41% |
| BLH | 0.68 ± 0.06 | 0% |
| **3T3 cells** | | |
| No GAG | 0.80 ± 0.05 | – |
| HS | 0.41 ± 0.04 | 49% |
| PMH | 0.59 ± 0.06 | 26% |
| **U2 cells** | | |
| No GAG | 0.27 ± 0.00 | – |
| HS | 0.02 ± 0.00 | 91% |
| PMH | 0.22 ± 0.03 | 17% |

[a] Cells were cultured in medium containing 10% FBS in the presence or absence of GAGs. After 72 h, cell number was determined by measuring the optical density (O.D.) ± standard deviation (S.D.) of cell preparations stained with crystal violet.
[b] GAGs were tested in triplicate at 167 μg/ml.

cell proliferation was not a result of cytotoxicity of the GAGs (data not shown).

*Effect of HS and PMH on proliferation of 3T3 fibroblasts and U-2 osteosarcoma cells:* To determine whether the growth inhibitory effects of HS and PMH were specific for SMC, the authors examined whether growth factor induced proliferation of other cell types could be suppressed by GAG treatment. As shown in Table 1, the growth of 3T3 fibroblasts was inhibited by the addition of either HS or PMH (167 μg/ml). It was also found that U-2 osteosarcoma cells were more sensitive to inhibition by HS (167 μg/ml) than NIH 3T3 cells or SMC.

*Inhibitory effect of HS on the proliferation of SMC in response to purified growth factors:* To confirm that the growth inhibitory effects of HS are effective on purified

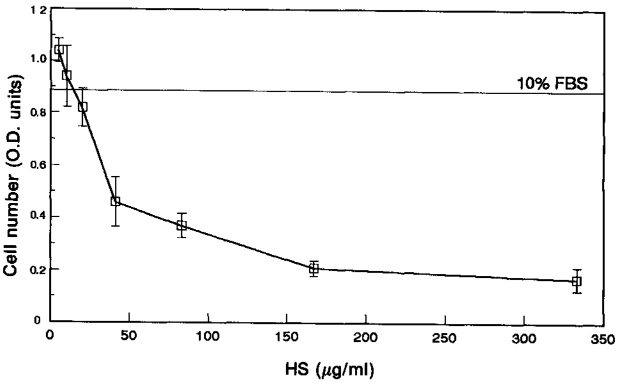

FIG. 1. Dose response of HS on Nor-10 SMC proliferation. Cells were treated with varying concentrations of HS as described in Materials and methods. O.D. was calculated as a mean of triplicate determinations ±S.D. 10% FBS line represents level of Nor-10 growth in the absence of GAG treatment.

**Table 2.** Effect of HS on TGFβ and PDGF induced Nor-10 proliferation

| Treatment[b] | Cell number[a] | |
|---|---|---|
| | O.D. ± S.D. | Inhibition |
| TGFβ alone | 0.65 ± 0.07 | – |
| TGFβ + HS | 0.31 ± 0.05 | 53% |
| PDGF alone | 0.42 ± 0.06 | – |
| PDGF + HS | 0.12 ± 0.03 | 72% |

[a] Cells were cultured in the presence or absence of growth factors with or without HS. After 72 h, cell number was determined by measuring the optical density (O.D.) ± standard deviation (S.D.) of cell preparations stained with crystal violet.
[b] GAGs were tested in triplicate at 167 μg/ml, TGFβ and PDGF were 15 ng/ml and 5 ng/ml, respectively.

growth factors, the regulatory effects of HS on the proliferation of SMC triggered by a TGFβ (15 ng/ml) and PDGF (5 ng/ml) (Table 2) was tested. The addition of HS (100 μg/ml) to cultures of Nor-10 cells given PDGF or TGFβ blocked the level of proliferation by 72% and 53%, respectively. This confirmed that the growth inhibitory effects of HS were not merely a phenomenological effect of serum administration or the adsorption of essential nutrients to the GAGs.

*Effects of limited exposure of SMC to HS on growth inhibition:* In an attempt to elucidate the mechanism of growth inhibition by HS, studies were aimed at determining whether HS acts by binding to growth factors and preventing their interaction with cell surface receptors or by exhibiting a direct effect on the target cell. To test this, SMC were cultured in the presence or absence of HS for 18 h after which, the medium was aspirated and replaced with medium containing growth factor (either 10% FBS or purified PDGF (2.5 ng/ml) in 1% FBS (Table 3)).

**Table 3.** Effect of pre-treatment of Nor-10 SMC with HS on proliferation induced by FBS or PDGF

| Treatment[b] | Cell number[a] | |
|---|---|---|
| | O.D. ± S.D. | Inhibition |
| FBS alone | 1.48 ± 0.06 | - |
| FBS + HS (3 μg/ml) | 1.15 ± 0.06 | 22% |
| (33 μg/ml) | 1.02 ± 0.10 | 31% |
| (167 μg/ml) | 0.13 ± 0.07 | 91% |
| PDGF alone | 0.36 ± 0.04 | - |
| PDGF + HS (167 μg/ml) | 0.15 ± 0.01 | 57% |

[a] Cells were cultured in the presence or absence of HS for 18 h after which the medium was replaced with medium containing growth factors (10% FBS or 5 ng/ml PDGF) without GAGs. Cells were incubated for an additional 48 h. Cell number was determined by measuring the optical density (O.D.) ± standard deviation (S.D.) of cell preparations stained with crystal violet.
[b] Proliferation was tested in triplicate.

The amount of inhibition induced by HS after 18 h of exposure ranged from 30% (33 μg/ml HS) to near 100% (167 μg/ml). Further experiments examined the effects of 18, 4, and 1 h pre-treatment of SMC. These experiments revealed that 4 h pre-treatment caused approximately 50% the inhibition seen at 18 h pre-treatment while 1 h pre-treatment had no inhibitory effect. Hence, the brief exposure of cells to HS (less than 4 h) did not halt the inhibitory activities of the GAG, suggesting that HS exhibits its effects by interaction with the target cell rather than blocking the interaction of growth factors with their receptors.

### Discussion

Glycosaminoglycans have been examined previously for their effects on cell proliferation.[9–12] A majority of these studies demonstrated that heparin moieties cause the greatest growth inhibition. The present authors, on the other hand, found an HS moiety that inhibits the proliferation of SMC, fibroblasts and an osteosarcoma cell line to a greater extent than any of the other GAGs tested, including heparin. Other HS moieties capable of inhibiting growth have been observed by other investigators. Reilly *et al.*[3] reported an HS species capable of inhibiting SMC growth. This HS was 40 times more active than heparin and was found to be uniquely placed on the cell surface of post-confluent SMC but not on exponentially growing cells, suggesting a possible role for HS as an endogenous mediator of proliferation. Recently, Benitz *et al.*[13] isolated an endothelial HS proteoglycan that was a potent inhibitor of SMC growth. The inhibitory activity of this HS was 1000 times greater than the tested heparin preparation. It is possible that there is a specific structural determinant of the HS used in both our studies and in these latter published studies, that confers a growth inhibitory property.

HS and heparin have certain structural similarities, including alternating D-glucuronic acid and *N*-acetyl-D-glucosamine units,[6,14] as well as similar biosynthetic pathways. Correlations between heparin's growth inhibitory activity and structure have been made previously.[1,15–17] Wright *et al.*,[15] examining the growth inhibitory activity of heparin on rat vascular smooth muscle cells (VSMC), calf VSMC, and rat cervical epithelial cells, found that hexasaccharide fragments were antiproliferative for all three cell types, while a synthetic pentasaccharide inhibited only the rat and calf VSMC. The largest anti-proliferative effects were observed with dodecasaccharide and larger fragments. An interdependence between size and charge was also observed. In addition, the degree of sulphation correlated positively with the anti-proliferative activity such that completely desulphated heparin failed to inhibit cell growth.[16] In contrast, the 2-O sulphate glucuronic moiety was found not to be required for antiproliferative activity.[12] Thus, sulphation and saccharide components may be critical elements in the anti-proliferative nature of GAGs.

Several contradictory studies have examined the growth modulatory interactions of GAGs and growth factors. Dupuy *et al.*[11] found that an unfractionated pig mucosal heparin (PMH) enhanced the growth inducing effects of PDGF on fibroblasts. This was also observed with FGF and EGF, but to a lesser extent. Dupuy and colleagues also noted that PDGF did not modify heparin binding or internalization, nor did it alter the interaction of PDGF with target cells. It has been reported that heparin and HS inhibited arterial SMC proliferation in the presence of PDGF.[4,18] The present results confirm and expand these findings with the observation that both HS and certain species of heparin block the proliferation of mesenchymal cells triggered by TGF$\beta$ and PDGF (Table 2).

Heparin binds to growth factors including FGF, endothelial cell growth factor, and PDGF.[19] The interaction of heparin and FGF prolongs the half-life of the growth factor, thus increasing its availability to cells.[20] Similar analyses have not yet been made with HS. The binding of heparin to growth factors may either prevent the growth factor from interacting with cells, or, alternatively, sequester it and present it to the cell thus prolonging its effects, as was reported for FGF. In contrast, our studies suggest that HS does not inhibit proliferation by binding to growth factors and preventing their interaction with cells. Even when permitted only brief exposure to HS (prior to treatment with serum or growth factors), the cells retained the growth inhibitory effect (Table 3). This is supported by the work of Reilly *et al.*[3] who found that heparin does not inhibit growth by preventing serum mitogens or nutrients from interacting with SMC. It is also possible that the HS is affecting cell growth by interfering with the expression or affinity of growth factor receptors. Some evidence suggests that heparin down-regulates EGF receptors on rat vascular SMC, but only when added late in the G1 phase of the cell cycle.[15] In contrast, the number of EGF receptors increases when rat cervical epithelial cells were treated with heparin. The modulation of growth factor receptor levels by HS has not been examined.

Further evidence supporting the direct interaction of GAGs with target cells includes the observation that heparin has specific, high affinity to cell surface receptors. Vascular SMC contain 100 000 binding sites per cell that bind heparin with a Kd of $10^{-9}$ M.[3,9] In addition, heparin may exhibit its inhibitory actions on BALB/c 3T3 cells by blocking the expression of growth factor inducible c-*fos* and c-*myc* expression. It is not known whether HS works in this way.

In conclusion, the authors have found a HS species which can inhibit the TGF$\beta$ and PDGF induced proliferation of a variety of cell types including Nor-10 SMC, 3T3 fibroblasts and U2-osteosarcoma cell lines. Further research on the molecular effects of HS on target cells will lead to an understanding of how HS inhibits cell growth. HS is not a strong anticoagulant, raising the possibility of its use as a therapeutic agent to prevent abnormal cell proliferation in diseases such as atherosclerosis and pulmonary fibrosis.

### References

1. Benitz W, Kelley R, Anderson C, Lorant D, Bernfield M. Endothelial heparan sulfate proteoglycan. I. Inhibitory effects on smooth muscle cell proliferation. *Am J Respir Cell Mol Biol* 1990; **2**: 13.
2. Castellot J, Favreau L, Karnovsky M, Rosenberg R. Inhibition of vascular smooth muscle cell growth by endothelial cell-derived heparin. *J Biol Chem* 1982; **257**: 11256.
3. Reilly C, Fritze L, Rosenberg R. Heparin inhibition of smooth muscle cell proliferation: a cellular site of action. *J Cell Physiol* 1986; **129**: 11.
4. Reilly C, Fritze L, Rosenberg R. Antiproliferative effects of heparin on vascular smooth muscle cells are reversed by epidermal growth factor. *J Cell Physiol* 1987; **131**: 149.
5. Reilly C, Kindy M, Brown K, Rosenberg R, Sonenshein G. Heparin prevents vascular smooth muscle cell progression through the $G_1$ phase of the cell cycle. *J Biol Chem* 1989; **264**: 6990.
6. Johnson E. Heparan sulphates from porcine intestinal mucosa. Preparation and physicochemical properties. *Thromb Res* 1984; **35**: 583.
7. Jaques L. Heparin: a unique misunderstood drug. *Trends Pharmacol Sci* 1982; **3**: 289.
8. Kamijo R, Takeda K, Nagumo M, Konno K. Suppression of TNF-stimulated proliferation of diploid fibroblasts and TNF-induced cytotoxicity against transformed fibroblasts by TGF-$\beta$. *Biochem Biophys Res Commun* 1989; **158**: 155.
9. Castellot J, Wong K, Herman B, *et al.* Binding and internalization of heparin by vascular smooth muscle cells. *J Cell Physiol* 1985; **124**: 13.
10. Castellot J, Cochran D, Karnovsky M. Effect of heparin on vascular smooth muscle cells. I. Cell metabolism. *J Cell Physiol* 1985; **124**: 21.
11. Dupuy E, Rohrlich P, Tobelem G. Heparin stimulates fibroblasts growth induced by platelet derived growth factor. *Cell Bio Int Rep* 1988; **12**: 17.
12. Wright T, Pukac L, Castellot J, Karnovsky M, Levine R, Kim-Park H, Campisi J. Heparin suppresses the induction of c-*fos* and c-*myc* mRNA in murine fibroblasts by selective inhibition of a protein kinase C-dependent pathway. *Proc Natl Acad Sci USA* 1989; **86**: 3199.
13. Benitz W, Lessler D, Coulson J, Barnfield M. Heparin inhibits proliferation of fetal vascular smooth muscle cells in the absence of platelet-derived growth factor. *J Cell Physiol* 1986; **127**: 1.
14. Lidholt K, Kjellen L, Lindahl U. Biosynthesis of heparin: relationship between the polymerization and sulphation processes. *Biochem J* 1989; **261**: 999.
15. Wright T, Castellot J, Petiou M, Lormeau J, Choay J, Karnovsky M. Structural determinants of heparin's growth inhibitory activity: interdependence of oligosaccharide size and charge. *J Biol Chem* 1989; **264**: 1534.
16. Castellot J, Wright T, Karnovsky M. Regulation of vascular smooth muscle cell growth by heparin and heparan sulfates. *Semin Thromb Hemost* 1987; **13**: 489.
17. Lippman M, Mathews M. Heparins: varying effects on cell proliferation *in vitro* and lack of correlation with anticoagulant activity. *Fed Proc* 1977; **36**: 55.
18. Fager G, Hansson K, Ottosson P, Dahllof B, Bondjers G. Human arterial smooth muscle cells in culture: effects of PDGF and heparin on growth *in vitro*. *Exp Cell Res* 1988; **176**: 319.
19. Vannucchi S, Pasquali F, Chiarugi V, Ruggiero M. Internalization and metabolism of endogenous heparin by cultured endothelial cells. *Biochem Biophys Res Commun* 1986; **140**: 294.
20. Damon D, Lobb R, D'Amore P, Wagner J. Heparin potentiates the action of acidic fibroblast growth factor by prolonging its biological half-life. *J Cell Physiol* 1989; **138**: 221.

ACKNOWLEDGEMENTS. This work was supported by ONR #N00014-89-J-1130 and the Elsa U. Pardee Foundation.

**Received 21 April 1993; accepted in revised form 13 May 1993**
